# Molecular Dynamics Simulation of High-Temperature Creep Behavior of Nickel Polycrystalline Nanopillars

**DOI:** 10.3390/molecules26092606

**Published:** 2021-04-29

**Authors:** Xiang Xu, Peter Binkele, Wolfgang Verestek, Siegfried Schmauder

**Affiliations:** Institute for Materials Testing, Materials Science and Strength of Materials, University of Stuttgart, Pfaffenwaldring 32, 70569 Stuttgart, Germany; peter.binkele@imwf.uni-stuttgart.de (P.B.); wolfgang.verestek@imwf.uni-stuttgart.de (W.V.); Siegfried.Schmauder@imwf.uni-stuttgart.de (S.S.)

**Keywords:** polycrystalline nanopillars, molecular dynamics method, creep mechanisms, dislocation creep, grain boundary sliding, deformation map

## Abstract

As Nickel (Ni) is the base of important Ni-based superalloys for high-temperature applications, it is important to determine the creep behavior of its nano-polycrystals. The nano-tensile properties and creep behavior of nickel polycrystalline nanopillars are investigated employing molecular dynamics simulations under different temperatures, stresses, and grain sizes. The mechanisms behind the creep behavior are analyzed in detail by calculating the stress exponents, grain boundary exponents, and activation energies. The novel results in this work are summarized in a deformation mechanism map and are in good agreement with Ashby’s experimental results for pure Ni. Through the deformation diagram, dislocation creep dominates the creep process when applying a high stress, while grain boundary sliding prevails at lower stress levels. These two mechanisms could also be coupled together for a low-stress but a high-temperature creep simulation. In this work, the dislocation creep is clearly observed and discussed in detail. Through analyzing the activation energies, vacancy diffusion begins to play an important role in enhancing the grain boundary creep in the creep process when the temperature is above 1000 K.

## 1. Introduction

Nanocrystalline (NC) metals possess many different mechanical behaviors in comparison with traditional coarse grained metals, e.g., the Hall–Petch effect and the inverse Hall–Petch effect, which have already been studied by several researchers [1,2,3,4]. For application at a high-temperature condition, the creep behavior is the most important one that should be carefully researched and thoroughly understood. The creep deformation in polycrystalline metals is well described by the Bird–Dorn–Mukherjee relation [5], given below as Equation (1).

(1) $$\dot{\varepsilon }=\frac{AD_{0}Gb}{k_{b}T}{\left(\frac{b}{d}\right)}^{p}{\left(\frac{\sigma }{G}\right)}^{n}\exp\left(-\frac{\Delta Q}{k_{b}T}\right)$$

In this equation, *A* is a dimensionless constant, $D_{0}$ is the diffusion constant (frequency factor), and *b* is the Burgers vector. *G*, $k_{b},$ and ΔQ are the shear modulus, the Boltzmann constant, and the activation energy for a thermally activated process, respectively. *p* represents the exponent of the grain size *d* and *n* the exponent of the applied stress σ. *T* is the temperature. With varying the temperature *T*, the applied stress σ, and the grain size *d* in a creep simulation, different creep behavior can be observed.

On the nanoscale, the diffusion process and the grain boundaries influence the creep mechanisms in a significant way, therefore many studies on this topic have already been published [6,7,8,9,10,11,12]. The exponent pair (n,p) for stress and grain size was used to determine the creep mechanisms. For example, n=1, p=2 was characterized as Nabarro–Herring creep (lattice diffusion) [10], and n=1, p=3 is Coble creep [6]. When n>3, the plastic deformation mechanism is dislocation creep, i.e., the power-law creep [13,14,15]. Moreover, Ashby introduced deformation maps that display the fields of stress and temperature in which a particular mechanism of plastic flow is dominant [16].

The development of molecular dynamics simulations provides a way to study the creep behavior of nanocrystalline materials. Swygenhoven et al. reported that the grain boundary sliding, motion, and orientation were responsible for the low-temperature high-load plastic deformation of the Ni nanophase [17]. The publications [18,19,20] approached creep behavior from the diffusion mechanism, the bending creep behavior, and the influence of porosity and voids on the creep behavior, respectively. Yamakov et al. studied the deformation mechanism, the nucleation and propagation of dislocations, the grain boundary diffusion, and the sliding of grain boundaries through MD simulations [21,22,23,24,25]. Moreover, addition of alloying elements also influences the creep behavior [26].

Because of the limitations of computer abilities, the grain size is restricted by the number of atoms inside the NC model. In previous works [12,13,14,27], the investigated grain sizes were not bigger than 16 nm, which led to a small free space for intra-grain dislocation movements. Hence, the discovered creep mechanisms were mostly limited to diffusion creep, grain boundary sliding, and dislocation nucleation.

Especially for Ni nanocrystals, Nie et al. [12] studied the creep behavior of NC Ni with consideration on temperatures, grain sizes, and stresses. The grain sizes in their work ranged from 2.8 to 5.6 nm. It was found that with increasing the temperature and stress level, the creep mechanisms changed from lattice diffusion and grain boundary sliding to grain boundary diffusion and then to dislocation nucleation. However, due to the limitation of grain sizes, the most important dislocation propagation and interactions were not studied.

This research took the temperature *T*, the applied stress σ, and the grain size *d* as variables and studied their influence on the creep behavior of Ni polycrystalline nanopillars. We chose grain sizes between 20–30 nm and discovered that the dislocations can interact with other dislocations or grain boundaries. Creep mechanisms were determined through stress exponents *n* and grain size exponents *p*. Corresponding mechanisms were also verified with the atomistic visualization of configurations dumped during the creep process.

## 2. Simulation Method and Process

Considering the accessible computer resources, our models contain approximately 10,000,000 atoms. All the models were first set up through the software Atomsk [28], using the implemented Voronoi method with random orientations and positions of crystalline seeds, which results in a nearly isotropic property for the model. After that, the models were simulated through the Large-scale Atomic/Molecular Massively Parallel Simulator (LAMMPS) [29]. An embedded atom method (EAM) potential Fe-Ni-Cu by Bonny [30] was used, in which the potential for Ni was adopted from Voter and Chen [31]. The elastic properties of Ni, computed with this potential, are summarized in Table 1. The atomistic visualization was carried out through the Open Visualization Tool (OVITO) [32].

### 2.1. Main Variables

**Figure 1:**
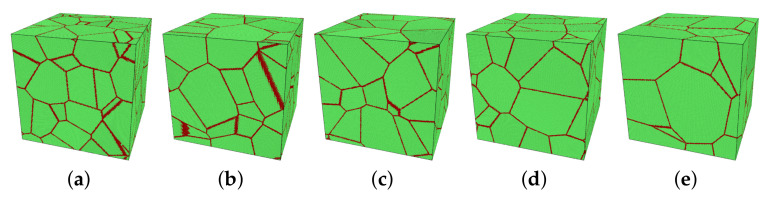
(**a**–**e**) Five models with different numbers of grains, named M1 to M5 for model 1 to model 5. These pictures are visualized by a polyhedral template matching (PTM) method [33] in OVITO (green: FCC; red: others as grain boundary). (**a**) M1: 30 grains, (**b**) M2: 25 grains, (**c**) M3: 20 grains, (**d**) M4: 15 grains, (**e**) M5: 10 grains.

In this study, the main variables were the mean grain size *d* of the model, the temperature *T*, and the applied stress σ. The initial size of simulation boxes was 50 × 50 × 50 nm^3^. The number of grains varied from 10 to 30 in five models, see Figure 1. Through approximating grains as spheres, the corresponding averaged diameter $\bar{d}$ can be calculated by $\bar{d}=\sqrt[{3}]{3V/\left(4\pi N\right)}$, in which *V* and *N* are the volume of the model and the number of grains in the model, respectively. Table 2 shows the grain size of each model.

The melting point $T_{m}$ of pure Ni was simulated in this work as 1720 K, which is close to the well-known value 1728 K. The investigated temperatures are 500, 800, and 1200 K for all five models. Additionally, in order to obtain more detailed information about the influence of temperature and to analyze the activation energy, the model M1 was simulated at a finer temperature mesh from 500 to 1200 K for every 100 K increment.

All the applied stresses in the creep simulations were homologous to the strength $R_{m}$. The applied stresses varied from 0.4 $R_{m}$ to 0.8 $R_{m}$.

### 2.2. Main Process of Simulations

The models were initially equilibrated at the selected temperatures, i.e., 500, 800, and 1200 K, as an isotherm-isobar (NPT) ensemble. Then the nano-tensile simulations were performed. The nano-creep simulations were sequentially executed with the applied uni-axial stresses level determined by the tensile strength. The applied boundary conditions of nano-tensile and -creep simulations were periodic in the x-direction and shrink-wrapped (not periodic but encompassing all the atoms inside the surface) in the y- and z-directions.

## 3. Results and Analysis of Simulations

### 3.1. Nano-Tensile Test Simulations

In order to obtain strengths $R_{m}$ of all models M1–M5, nano-tensile tests at different temperatures were carried out prior to nano-creep simulations.

**Figure 2:**
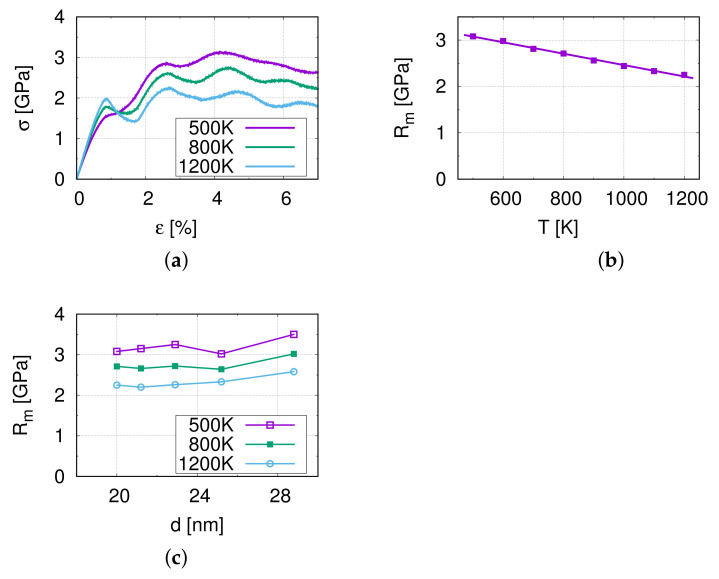
The results of nano-tensile simulations. (**a**) Stress–strain curves at the corresponding temperatures for M1. (**b**) The temperature dependence of the tensile strength $R_{m}$. (**c**) The influence of the grain size on the tensile strength $R_{m}$.

Figure 2a shows the stress–strain curves of M1 at different temperatures. At the beginning, it is a linear elastic region, and the slope of this section (ε to 0.5%) is the Young’s modulus, 221.25 GPa at 500 K. Compared with Table 1, we can see that the difference is very small. This indicates that the model is almost isotropic.

In Figure 2a an oscillation can be observed of the resulting tensile stress in the stress–strain curve with the frequency varying from 14 to 18 ps (strain from 1.4 to 1.8%). This oscillation is considered to be caused by the non-periodic boundary conditions in the y- and z-directions in combination with the elastic pulse when starting the tensile simulation. Furthermore, the maximum amplitude at the first cycle at a strain of approximately 0.9% shows a higher stress at 1200 K than at 800 K and 500 K, and indicates a temperature influence on this effect. Further analysis shows that the oscillation decays rapidly and therefore, the influence of the oscillation on the ultimate tensile strength is minor.

Figure 2b,c display the temperature influence on the tensile strength of M1. The tensile strength of model M1 decreases from 3.08 to 2.25 GPa as the temperature increases, which is due to the softening of the material at higher temperatures. Figure 2c demonstrates the relation between the grain size and the tensile strength $R_{m}$. The tensile strength shows no significant dependence on the grain size in the investigated range. This suggests that the relevant grain sizes lie around the transition regime between the Hall–Petch and the reverse Hall–Petch effect.

### 3.2. Nano-Creep Simulations

**Figure 3:**
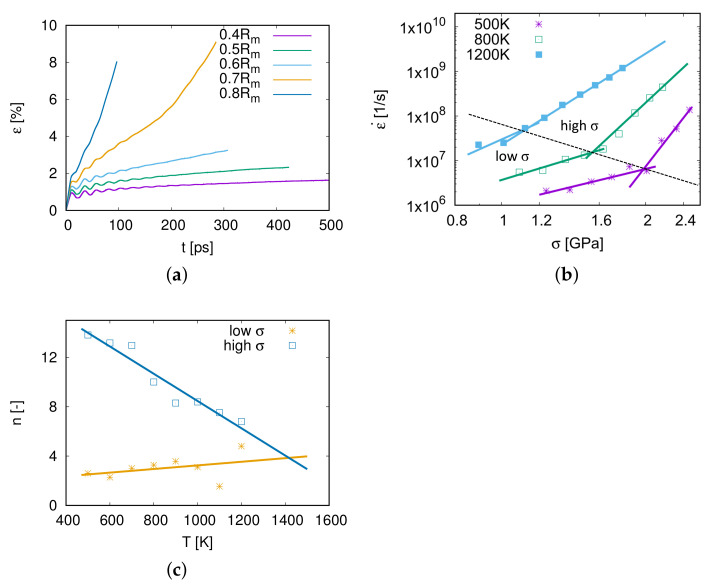
(**a**) Creep curves of model M1 for different stresses at 1000 K. (**b**) The log–log scaling plot of creep rates $\dot{\varepsilon }$ over σ at different temperatures. The data are split up into a low σ region and a high σ region and were fitted with different exponents *n*. (**c**) The relationship between the stress exponent *n* and temperature.

As shown in Figure 3a, the creep strain increases with the simulation time. At the beginning of the simulation, there are some fluctuations of the creep strain, because the stress was applied to the model within a short time interval. The second phase of the creep process is steady and linear, and the creep rate $\dot{\varepsilon }=d\varepsilon /dt$ in this phase is the minimum creep rate during the whole process. Under an applied stress σ=0.7$R_{m}$, the creep process of model M1 steps into the third phase at 200 ps. When the applied stress increases to 0.8 $R_{m}$, the second phase is very short and the creep process steps directly into the third phase. The second creep phase, which can be clearly observed in this work, plays the most important role during the creep process. Therefore, the simulation time around 500 ps is sufficient to investigate the dominant creep mechanisms.

The minimum creep rate $\dot{\varepsilon }$ is of great importance to the creep property. In Figure 3b, the relation of $\dot{\varepsilon }$ and σ at different temperatures *T* is revealed in a log–log scaling diagram. The higher the applied stress is, the faster the model creeps. Furthermore, it can also be seen from Figure 3b that the creep rate $\dot{\varepsilon }$ increases with temperature.

According to the power-law relationship of the strain rate $\dot{\varepsilon }$ and the applied stress σ from the Bird–Dorn–Mukherjee relation (Equation (1)), the stress exponent is expressed as $n=\partial \log\dot{\varepsilon }/\partial \log\sigma$. *n* is the slope in the log–log scaling plot of $\dot{\varepsilon }$ with σ. As displayed in Figure 3b, the relationship between $\log\dot{\varepsilon }$ and $\log(\sigma /R_{m})$ is not linear. Therefore the data were cut into two regions, a low σ region and a high σ one, and these calculated exponents are shown in Figure 3c and in Table 3.

From Figure 3c and Table 3, it is considered that when applying high stresses at a temperature between 500 and 1200 K, the dominant mechanism is the power-law creep as is well-known as the dislocation creep because of stress exponents n>3.

For creep tests at low stresses, stress exponents are larger than 2 and increase with temperature. When T<700*K*, the creep mechanism for low stresses is distinguished as grain boundary sliding as the stress exponent 2.6<n<3 [12]. When the temperature is above 800 K, the creep mechanism is supposed to be coupled by grain boundary sliding and dislocation nucleation and propagation.

The yellow line fitted with stress exponents of low σ regions shows a deviation for the two points at 1100 and 1200 K. This is due to fewer data points for fitting low stress exponents at a high temperature, e.g., 6 points at 500 K but 3 points at 1200 K in Figure 3b. However, the yellow line can still provide an approximate prediction of the stress exponent *n* of the low σ region for higher temperature conditions.

It is interesting to emphasize that the stress exponents of low- and high-stress parts have an intersection at around (1400 K, n=4), as shown in Figure 3c. This means that the creep mechanism above 1400 K is independent of the stress ranging from 0.4 $R_{m}$ to 0.8 $R_{m}$, and the stress exponent n=4 represents that the grain boundary sliding is coupled with dislocation slip (see Section 4.3).

### 3.3. Thermally Activated Mechanisms

**Figure 4:**
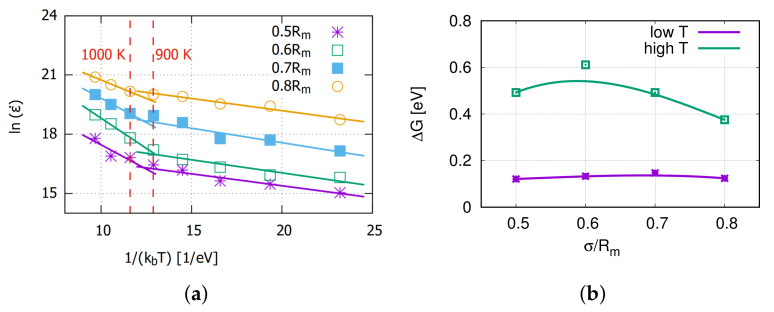
(**a**) The plot of $\ln\dot{\varepsilon }$ versus $1/(k_{B}T)$, which is derived from the Arrhenius equation. (**b**) Corresponding activation energies.

The Arrhenius equation was applied here to analyze the temperature influence on the creep rate. Figure 4 shows the data and fitted lines for $\ln\dot{\varepsilon }$ and $1/(k_{b}T)$, which are derived from the Arrhenius equation. The slope of every fit line is the free activation energy ΔG of the creep process.

From Figure 4a, the creep rate at a certain stress can be divided into two different temperature ranges. The turning point is around 900∼1000 K for all applied stresses. It is assumed to be the thermal activation for the vacancy diffusion as Aidhy et al. [34] reported that the vacancies are immobile up to 700 K and diffusing at 1200 K. At high temperatures, the activation energy for the accelerated creep process is around 0.5 eV, which is slightly lower than the 0.8 eV reported by Swygenhoven [18]. This might be caused by a couple mechanism with grain boundary diffusion or dislocation gliding.

## 4. Discussion on Creep Mechanisms

### 4.1. Deformation Diagram for NC Ni

Although the creep rate in the MD simulation is of around 8 orders of magnitude faster than in experimental results, which is the typical timescale and/or length scale problem for MD simulations, the simulation results reveal the mechanism of plastic deformations when we normalize the parameters to dimensionless.

**Figure 5:**
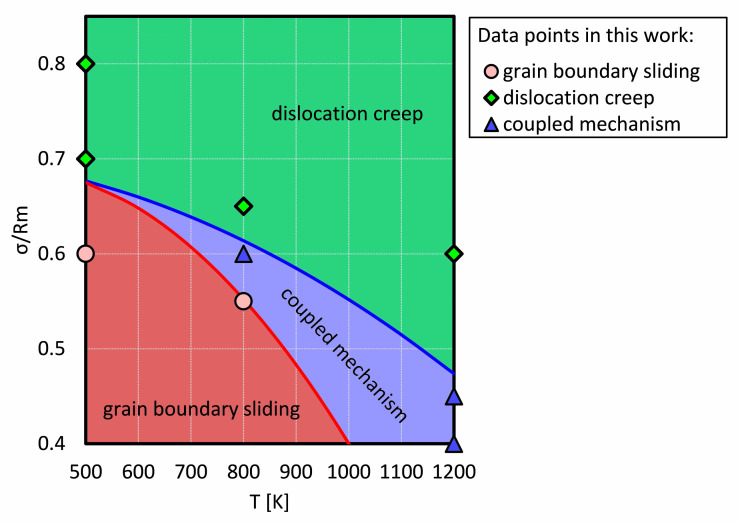
The schematic plastic deformation map created in this work. The applied stress was normalized to the tensile strength. The coupled mechanism means the creep mechanism is coupled by dislocation creep and grain boundary creep.

In order to compare our work with experimental results, all plastic deformations were collected and a deformation diagram for NC Ni was drafted in this work, as shown in Figure 5. The applied stresses were normalized to the corresponding tensile strength $R_{m}$ at each temperature. The green, blue and red regions were divided by certain (σ,T) points as investigated in this work, which represent grain boundary sliding, dislocation creep, and their coupling, respectively.

It is clear that at a low temperature and with a low stress, the creep process is dominated by grain boundary sliding, because the dislocation is difficult to nucleate at grain boundaries and to propagate into the grain. However, at a high stress level, dislocation nucleation and gliding is the dominant mechanism.

There is a common area (the blue area in Figure 5) where both dislocations and grain boundary sliding mechanisms become visible and make comparable contributions to the creep process. However, the red and green areas depict that a mechanism is dominant (but not exclusive). We propose that the transition from a grain boundary sliding dominated creep to a dislocation dominated creep is narrow at a lower temperature, e.g., the distance between the blue and the red line is smaller at 700 K than at 800 K. The conjunction point at around 0.68 $R_{m}$ approximately represents that the transition between grain boundary sliding and dislocation creep is rarely observable.

However, for a creep process with a coupled mechanism, to accurately determine the contribution of each mechanism is rather difficult. Hence, the region of the coupled mechanism is to represent that the grain boundary sliding and dislocation creep possess a comparable contribution to a creep process, which will be discussed in Section 4.3.

**Figure 6:**
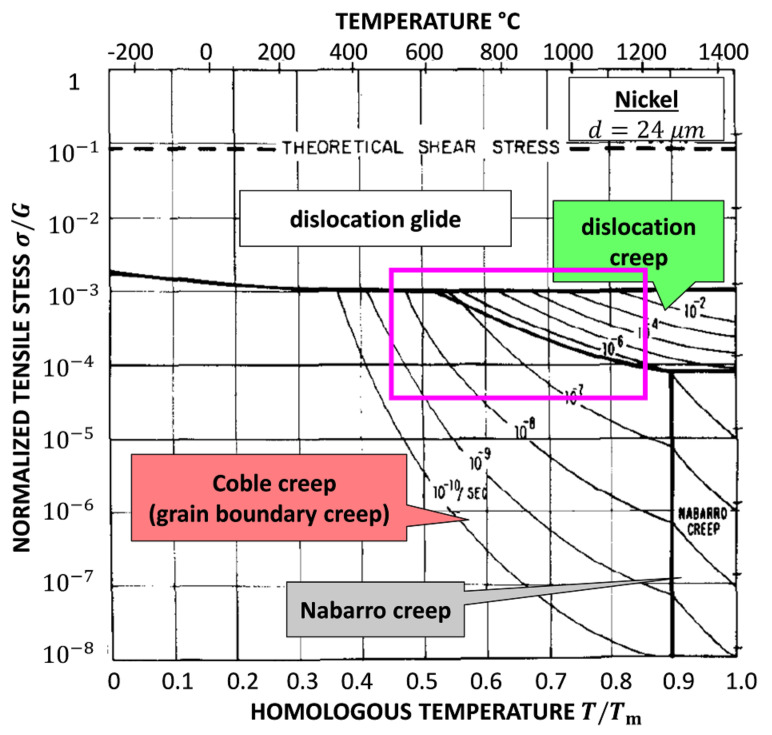
The Ashby map for plastic deformation of pure Ni with the grain size of 32 µm [16]. The area in the magenta box coincides with this work as shown in Figure 5.

Figure 6 is the famous Ashby map for Ni with a grain size of 32 µm [16]. The area in the magenta box shows the region of interest with mechanisms found in this work. It is significant that our result is comparable to the Ashby map. In this work, the lowest normalized stress $\sigma /G=1.16\times 10^{-2}$ for 0.4 $R_{m}$ at 1200 K is still higher than the stress corresponding to dislocation creep and grain boundary creep in the Ashby map. Because of the size effect, the yield strength and tensile strength are almost 7 times higher than normal results, e.g., 368 MPa at 973.15 K [35]. This is presumed to be the reason why a comparable deformation diagram is obtained at a higher stress level.

As we have already discussed the tensile simulation in Section 3.1, the main parts of this section are dedicated to dislocation creep, grain boundary sliding, and their coupling.

### 4.2. Creep through Dislocations

**Figure 7:**
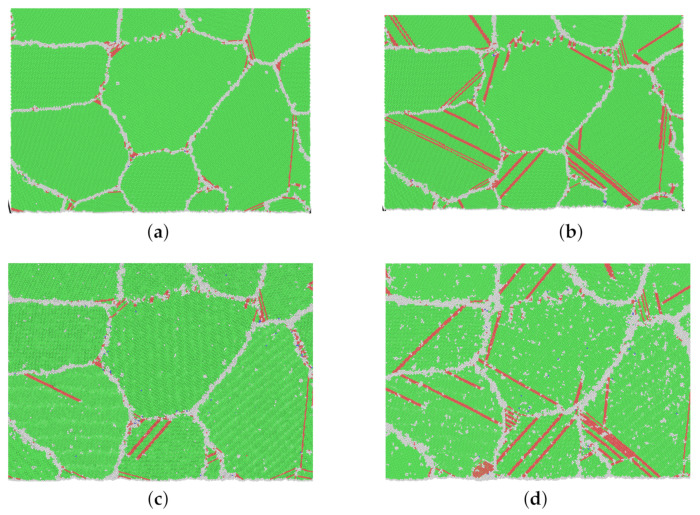
(**a**–**d**) Snapshots of atomic-scale crystalline structures of model M1 at 100 ps in different creep processes. (Green: FCC; red: stacking fault; gray: other.) (**a**) 500 K–0.4 $R_{m}$–100 ps, (**b**) 500 K–0.8 $R_{m}$–100 ps, (**c**) 1200 K–0.4 $R_{m}$–100 ps, (**d**) 1200 K–0.65 $R_{m}$–100 ps.

In Figure 7, snapshots of model M1 from different creep processes at the moment of 100 ps are shown. It is significant that at a higher stress level (refer to the green area in Figure 5), many more dislocations and stacking faults appear. From Figure 7c for 1200 K and 0.4 $R_{m}$ and Figure 7d for 1200 K and 0.65 $R_{m}$, intensive grain boundary sliding is also observed.

Referring to Table 3, the calculated stress exponents *n* for high stress are 13.8 and 6.8 for 500 and 1200 K, respectively. Combined with Figure 7, it is in no doubt that the dislocation nucleation and propagation dominate the creep process at a high stress level.

**Figure 8:**
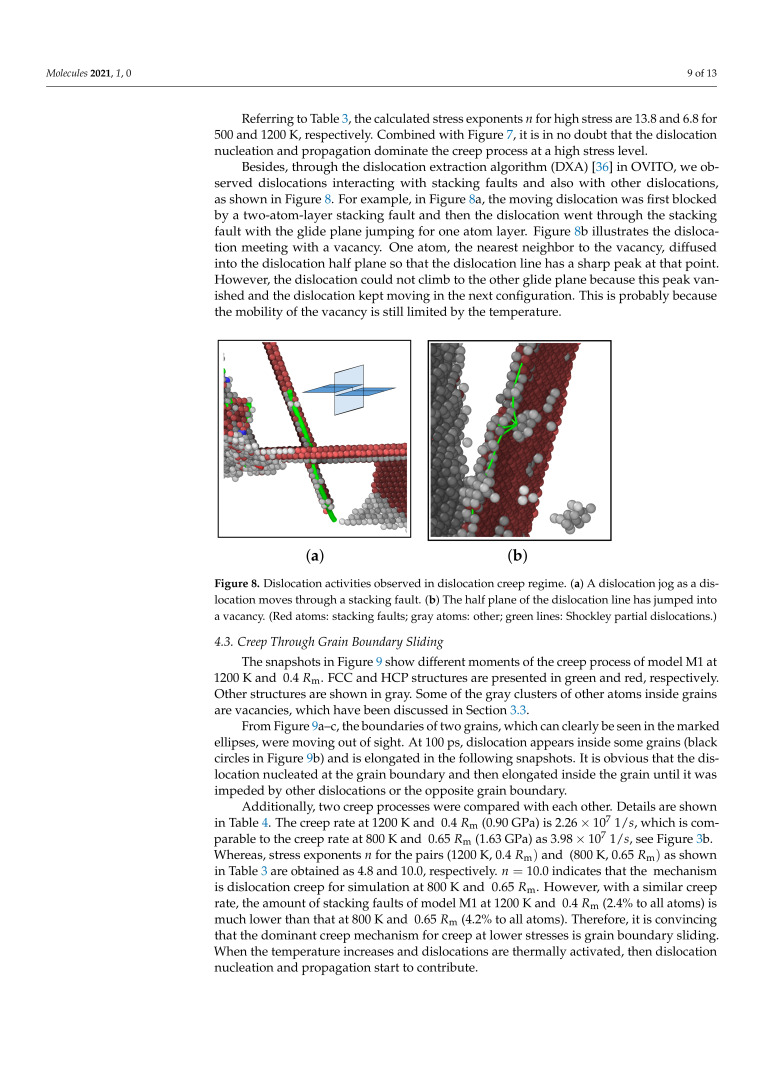
Dislocation activities observed in dislocation creep regime. (**a**) A dislocation jog as a dislocation moves through a stacking fault. (**b**) The half plane of the dislocation line has jumped into a vacancy. (Red atoms: stacking faults; gray atoms: other; green lines: Shockley partial dislocations.)

Besides, through the dislocation extraction algorithm (DXA) [36] in OVITO, we observed dislocations interacting with stacking faults and also with other dislocations, as shown in Figure 8. For example, in Figure 8a, the moving dislocation was first blocked by a two-atom-layer stacking fault and then the dislocation went through the stacking fault with the glide plane jumping for one atom layer. Figure 8b illustrates the dislocation meeting with a vacancy. One atom, the nearest neighbor to the vacancy, diffused into the dislocation half plane so that the dislocation line has a sharp peak at that point. However, the dislocation could not climb to the other glide plane because this peak vanished and the dislocation kept moving in the next configuration. This is probably because the mobility of the vacancy is still limited by the temperature.

### 4.3. Creep Through Grain Boundary Sliding

**Figure 9:**
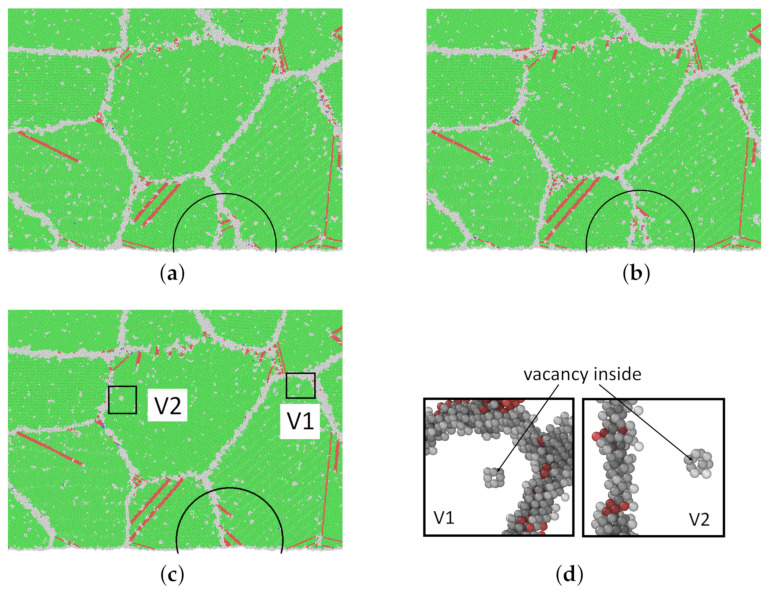
(**a**–**c**) Snapshots of atomic-scale crystalline structures of model M1 at 1200 K and $0.4R_{m}$ at different times in the creep process. The grain depicted in the half circle was moving out of sight during the creep process from 100 to 200 ps. (**d**) The nearest neighbor atoms of two vacancies that are enlarged from (**c**). For a clear illustration, the FCC atoms are deleted in (**d**). (Green: FCC; red: stacking fault; gray: others.) (**a**) 100 ps; (**b**) 150 ps; (**c**) 200 ps; (**d**) two vacancies.

The snapshots in Figure 9 show different moments of the creep process of model M1 at 1200 K and 0.4 $R_{m}$. FCC and HCP structures are presented in green and red, respectively. Other structures are shown in gray. Some of the gray clusters of other atoms inside grains are vacancies, which have been discussed in Section 3.3.

From Figure 9a–c, the boundaries of two grains, which can clearly be seen in the marked ellipses, were moving out of sight. At 100 ps, dislocation appears inside some grains (black circles in Figure 9b) and is elongated in the following snapshots. It is obvious that the dislocation nucleated at the grain boundary and then elongated inside the grain until it was impeded by other dislocations or the opposite grain boundary.

Additionally, two creep processes were compared with each other. Details are shown in Table 4. The creep rate at 1200 K and 0.4 $R_{m}$ (0.90 GPa) is $2.26\times 10^{7}$1/s, which is comparable to the creep rate at 800 K and 0.65 $R_{m}$ (1.63 GPa) as $3.98\times 10^{7}$1/s, see Figure 3b. Whereas, stress exponents *n* for the pairs (1200 K, 0.4 $R_{m})$ and (800 K, 0.65 $R_{m})$ as shown in Table 3 are obtained as 4.8 and 10.0, respectively. n=10.0 indicates that the mechanism is dislocation creep for simulation at 800 K and 0.65 $R_{m}$. However, with a similar creep rate, the amount of stacking faults of model M1 at 1200 K and 0.4 $R_{m}$ (2.4% to all atoms) is much lower than that at 800 K and 0.65 $R_{m}$ (4.2% to all atoms). Therefore, it is convincing that the dominant creep mechanism for creep at lower stresses is grain boundary sliding. When the temperature increases and dislocations are thermally activated, then dislocation nucleation and propagation start to contribute.

### 4.4. Grain Size Effect

**Figure 10:**
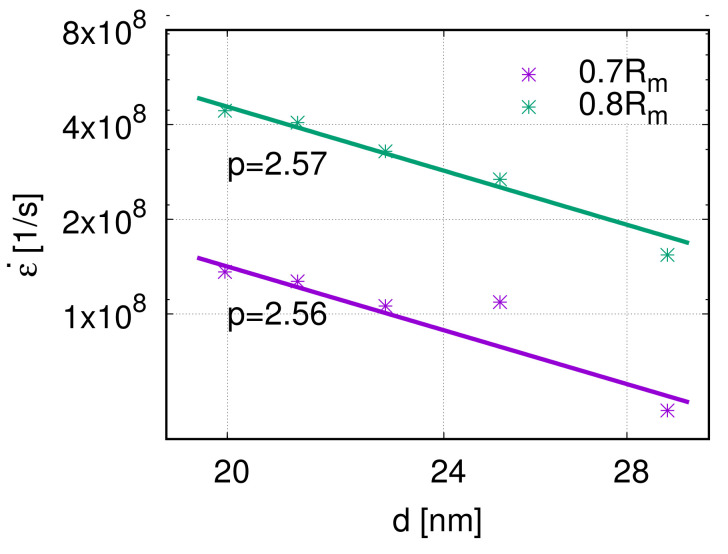
Log–log scaling plot of creep rate $\dot{\varepsilon }$ over grain size *d* and the fit-curve at 800 K. The grain size exponents are p=2.57 at 0.7 $R_{m}$ and p=2.04 at 0.8 $R_{m}$, respectively.

The grain size is the third factor that influences the creep properties. According to Equation (1), the grain size exponent can be calculated as $n=\partial \left(\log\dot{\varepsilon }\right)/\partial \left(\log(1/d)\right)$. The grain size exponents *p* of the five models are 2.56 and 2.57, respectively, at the stress levels 0.7 $R_{m}$ and 0.8 $R_{m}$, see Figure 10. This means that, at a high stress level, the creep rate decreases with increasing grain size. Because of the limitation of grain size researched in previous works, dislocation nucleation was observed and analyzed. However, the interaction between dislocations inside the grain has not been studied in a nano-creep simulation. Around the transition regime between the Hall–Petch and inverse Hall–Petch effects, the grain size has a sufficient influence on the dislocation movement, which is closely related to the mechanical properties. Therefore, it is not trivial to investigate further in this direction.

## 5. Summary

Through MD-simulations, we first studied the tensile properties of Ni nano-polycrystals. As the temperature decreased, the tensile strength $R_{m}$ increased. The tensile strength showed no significant dependence on the grain size. This might be due to the fact that the investigated grain sizes lie in the transition area between the Hall–Petch and inverse Hall–Petch regimes.

With applying stress that was homologous to the tensile strength $R_{m}$, nano-creep simulations were performed at different temperatures for all five models. From the creep curves, three typical creep phases have been clearly identified. We draw the conclusion that the creep rate rises with increasing stress, increasing temperature, and decreasing grain size. Collecting all plastic deformation cases, we formed a deformation mechanism map to distinguish the corresponding mechanisms at given conditions.

When applying a high stress, the stress exponent *n* was above 6.7, resulting in a dislocation creep. Visualization analysis revealed many dislocation movements, including nucleating from the grain boundary, propagating inside grains, and interacting with other dislocations or with grain boundaries.

As for a creep simulation at a low stress level, the dominant creep mechanism is supposed to be grain boundary sliding at a low temperature with stress exponents n<3. When increasing the temperature to 1200 K, the stress exponent for the low stress part increases to 4.8 and dislocations begin to contribute to the creep process. Hence, it is safely concluded that the dominant creep mechanism is grain boundary sliding at low stress and this could be coupled with dislocation creep with increasing the temperature. Additionally, from the analysis of activation energies, it was found that the vacancy diffusion becomes prevalent when the temperature is above 1000 K. The grain boundary creep is assumed to be enhanced by vacancy diffusion at high temperatures. Furthermore, we postulate that the creep mechanism of NC Ni remains unchanged with the coupling of dislocation creep and grain boundary creep when the temperature is above 1400 K.

It is novel that the deformation diagram corresponds well to the Ashby map for pure Ni. Due to the properties of NC metals, it is difficult and expensive to experimentally investigate the creep behavior of NC metals at high temperatures. This work could serve as a good example to expand deformation diagrams for NC metals through employing molecular dynamics simulations.

## Figures and Tables

**Table 1:** The modulus of pure Ni at different temperatures.

Temperature	Bulk Modulus	Poisson’s Ratio	Young’s Modulus	Shear Modulus
*T* [K]	*K* [GPa]	μ [-]	*E* [GPa]	*G* [GPa]
500	162.754	0.379	223.330	95.427
800	150.985	0.379	207.814	88.310
1200	135.164	0.381	189.529	77.88

**Table 2:** The averaged grain size *d* of every model.

Model	Number of Grains *N*	Averaged Grain Size *d* [nm]
M1	30	19.96
M2	25	21.22
M3	20	22.85
M4	15	25.15
M5	10	28.79

**Table 3:** The stress exponents *n* for different stresses and temperatures.

	500 K	600 K	700 K	800 K	900 K	1000 K	1100 K	1200 K
low σ	2.6	2.3	3.0	3.3	3.6	3.1	1.5	4.8
high σ	13.8	13.2	13.0	10.0	8.3	8.4	7.5	6.8

**Table 4:** Comparison of two creep processes. The stacking fault ratio represents how many are atoms with an HCP structure compared with the total number of atoms in the model.

Temperature [K]	800	1200
stress level	0.65 $R_{m}$	0.4 $R_{m}$
stress [GPa]	1.76	0.90
creep rate [1/s]	$3.98\times 10^{7}$	$2.26\times 10^{7}$
stress exponent *n*	10.0	4.8
stacking fault ratio at 100 ps	4.3%	2.4%

## Data Availability

The data presented in this study are available on request from the corresponding author. The data are not publicly available due to the ongoing research.
